# Characteristics associated with medication adherence in a randomized clinical trial of multiple pharmacotherapy adaptations based on treatment response in black adults who smoke

**DOI:** 10.1016/j.addbeh.2025.108431

**Published:** 2025-07-11

**Authors:** Erica Cruvinel, Alexandra Brown, Amanda Pritchard, Matthew S. Mayo, Lisa Sanderson Cox, Eleanor L.S. Leavens, Nicole L. Nollen

**Affiliations:** a Department of Population Health, University of Kansas Medical Center, Kansas City, KS 66160, United State; b Department of Biostatistics & Data Science, University of Kansas Medical Center, Kansas City, KS 66160, United States

**Keywords:** Smoking cessation, Pharmacotherapy, Treatment adherence and compliance, Clinical trial, Black people

## Abstract

**Introduction:**

Pharmacotherapy is a key component of evidence-based smoking cessation treatment and a major predictor of success in quitting. However, most people attempting to quit in clinical trials fail to fully adhere to their pharmacological treatments.

**Aims and methods:**

To assess factors associated with adherence to smoking cessation pharmacotherapies among Black adults participating in a clinical trial and to understand reasons for non-adherence to treatment. Data came from 333 participants enrolled in a randomized clinical trial of adapted therapy (ADT) or enhanced usual care (UC) for smoking cessation. Medication adherence was defined as taking at least 80 % of the prescribed medication for the entire treatment. Characteristics associated with 18-week adherence included demographic, psychosocial, smoking characteristics, substance use, medication experience, early treatment response, and adverse events. A best-subsets regression analyses were performed with all characteristics, and descriptive statistics were used to summarize the reasons for non-adherence to medication.

**Results:**

One hundred seventy-seven participants were compliant with their medication. Participants randomized to the ADT (OR = 2.40, 95 % CI = 1.52–3.81, p < 0.001) and with more positive medication experience (OR = 1.28, 95 % CI = 1.14–1.45, p < 0.001) were more likely to be adherent to study medications. The most common reason for non-adherence was “forgetting or losing the medication” (49.1 %).

**Conclusions:**

Over half of our sample were adherent to their pharmacotherapy, and those randomized to adaptive therapy, coupled with a positive experience with their medication, exhibited better overall compliance with their long-term pharmacotherapy. Reasons for not taking medications were similar to the existing literature.

## 1. Introduction

Pharmacotherapy and counseling are key components of evidence-based smoking cessation treatment and major predictors of success in quitting (Fiore & Baker, 2008). Nicotine replacement therapy (NRT), bupropion and varenicline all increase the chance of quitting compared to placebo. Specifically, NRT and bupropion almost double the quit rates among Black smokers (21.5 % versus 13.7 % and 36.0 % versus 19.0 %, respectively) (Robles et al., 2008) while varenicline nearly triples the likelihood of abstinence (OR = 2.77) compared to placebo (Cox, 2022).

Nevertheless, the majority of people who smoke and attempt to quit in clinical trials fail to fully adhere to their prescribed pharmacological treatments (Pacek et al., 2018). For instance, adherence with the recommended length of treatment occurs in no more than 50 % of NRT (Burns and Levinson, 2008) and varenicline users (Cox, 2022). In the main outcome paper from our clinical trial evaluating the efficacy of multiple pharmacotherapy adaptations (Nollen, 2023), we reported the overall adherence rates across 12 weeks of treatment, defined as taking 80 % or more of the prescribed doses across all visits, and found that 59.7 % of patients were adherent in the adaptive treatment group compared to 55.6 % in the usual care group. The lack of compliance with the recommended treatment length is significant because substantial evidence indicates that greater medication adherence is associated with greater abstinence from smoking, although the relationship is not causal (Bauer et al., 2021). Identifying factors that influence medication adherence could inform strategies to enhance engagement with smoking cessation pharmacotherapy. However, correlates of adherence to pharmacological interventions are often unreported in the literature (Pacek et al., 2018). The goal of this paper was to assess factors associated with medication adherence among Black participants in a smoking cessation clinical trial and to understand reasons associated with low adherence to pharmacological treatment.

## 2. Methods

### 2.1. Procedures

This is a secondary analysis of data from an unblinded and open-label randomized clinical trial conducted at the University of Kansas Medical Center (KUMC) to evaluate the efficacy of multiple pharmacotherapy adaptations in combination with counseling for smoking cessation among Black adults (Nollen, 2023). All participants received an FDA-approved medication for tobacco use: either bupropion, varenicline, or transdermal nicotine patches (NP). Participants randomized to enhanced usual care (UC) received 18-weeks of 24-hour 21 mg NP. Participants randomized to adapted therapy (ADT) received 2 weeks of 24-hour 21 mg NP at baseline and up to two pharmacotherapy adaptations, with a first switch to varenicline and a second switch, if needed, to bupropion + NP based on CO-verified smoking status (CO ≥ 6 ppm) at weeks 2 and 6.

### 2.2. Participants

All participants from the parent trial who completed the 18-week follow-up visit (N = 333/392) were included. All participants were from Missouri or Kansas, non-Hispanic Black adults (≥18 years), daily smokers and interested in quitting smoking when enrolled in the trial. The full protocol and main outcomes have been published elsewhere (Nollen, 2022, 2023). Study procedures were approved and monitored by the KUMC Institutional Review Board.

### 2.3. Measures

#### 2.3.1. Primary outcome: medication adherence

Medication adherence was assessed via 7-day Timeline Follow Back Interview at each in-person visit and corroborated with patch and pill counts. Participants were asked to return unused medications to ensure the correct counting of medications remaining in the pill box or patch dispensing bags. Adherence was defined as taking 80 % or more of the prescribed doses at each of the 2-, 6-, 12-, and 18-week follow-up visits. Using this stringent approach, participants must have taken at least 80 % of the study medication at all follow-up visits to be considered ‘adherent.’ Rates of adherence over time by treatment arm, including by treatment within the ADT arm are reported in the eTable1 of the main outcomes paper (Nollen, 2023). The adherence rates differed by treatment arm and medication, ranging from 59.6 % to 73.1 % at week 2 to 27.6 % to 76.5 % by week 12. We refer the reader to the main outcomes paper for this information (eTable1, page 3).

#### 2.3.2. Characteristics associated with non-adherence

The study baseline surveys collected several measures that are known to be robust determinants of tobacco use and cessation, including age, sex assigned at birth, employment status, the highest level of education, health insurance status, estimated yearly household income, housing status, and tobacco use characteristics. Tobacco variables included the number of cigarettes smoked in the past seven days, use of mentholated or non-mentholated cigarettes, smoke with 30 min of waking, urinary cotinine and 3-hydroxycotinine/cotinine, depression (Kroenke et al., 2003) and generalized anxiety symptoms (Spitzer, 2006), perceived stress (Cohen et al., 1983), perceived discrimination (Sternthal et al., 2011) neighborhood disadvantage (Feldman and Steptoe, 2004) and adverse childhood experiences (Felitti, 2019).

#### 2.3.3. Follow-up measures

This included any marijuana use or use of other tobacco products in the past seven days, measured at baseline and Weeks 2, 6, 12, and 18, as well as past 30-day prescription pain reliever use, measured at baseline and Weeks 6, 12, and 18. Those who self-reported marijuana, other tobacco products or pain reliever use at any assessment time point were categorized as ever user, while those who self-reported no use at all time points were categorized as non-user. Any adverse event experience was measured at weeks 2, 6, and 12. Participants who reported side effects at any assessment time point were categorized as experiencing side effects. Experiences with medication [assessed using a scale of 0 to 10, with 0 being ‘extremely negative’ and 10 being ‘extremely positive on the following question “*How would you rate your experience with this medication so far*?”] were averaged across the 2, 6, and 12-week time points. Abstainers at 2 weeks was defined as someone who reported no cigarette use in the past 7 days verified with a CO ≤ 6 ppm measured at week 2.

#### 2.3.4. Reasons for non-compliance to medication

We asked participants about primary reasons for not taking or partially taking their smoking cessation medications during the 2, 6, and 12-week follow-up visits using a closed question. Response options included “*quit smoking and didn’t need it”, “changed my mind about quitting”, “wanted to quit without medication”, “personal beliefs about this medication”, “a family member, friend, health care professional or someone else I care about expressed concern about this medication”, “forgot or lost the medication”, “study team discontinued medication due to adverse event,” or “other*”. ‘Other’ responses were recoded into one of the previous categories or new themes were created for commonly occurring responses. EC initially created a list with other responses and suggested codes. Then, a second investigator (NN) used this list to devise categories/definitions for those responses.

### 2.4. Data analysis

Differences in baseline and follow-up covariates between participants adherent and non-adherent were examined using means, frequencies, and appropriate statistical tests. The data analysis involved a series of logistic regressions, with medication adherence as the outcome measure. Best-subsets logistic regression in SAS 9.4 (SAS Institute, Cary, NC) was performed on significant variables from the univariate analysis to determine the best fitting model with the baseline and follow-up measures that were significantly associated with week 18 adherence. The final model was selected based on its ability to discriminate between outcomes, as measured by the c-statistic, Somers’ D, and the percent concordant. We performed a completer-only analysis, meaning those who participated in their Week 18 visit. If any interim visits were missed but the week 18 visit was observed, these participants were considered non-adherent. However, there were very few observations imputed to non-adherent across follow-up visits and rates of imputation for non-adherence did not differ by treatment [UC: 13 (7.7 %) vs ADT: 5 (3.1 %), p = 0.06]. Sensitivity analyses excluding these 18 participants did not change the results. We also explored baseline differences (age, income, cigarettes per day, menthol use, sex, education) between the 333 participants that completed Week 18 visit and the 59 who did not. We found the participants that completed the Week 18 visit were older and had lower income, all other factors were similar between the two groups. Additional descriptive analyses were performed to investigate reasons for non-adherence to medication. We analyzed responses from participants who were categorized as partially or never compliant with the prescribed treatment. We analyzed the response frequency at each time point to identify reasons for non-compliance throughout the 12-week treatment period.

## 3. Results

More than half of the sample were compliant with their prescribed medication (N = 177/333, 53.1 %) over 18 weeks of treatment. Table 1 shows the sample characteristics of participants who were non-adherent and adherent to study medications. Groups were similar in most characteristics, differing only in the housing situation, with significantly more participants in the non-adherent group owning a home (21.8 % vs 12.4 %), more people in the adherent group smoking 30 min of waking (84.2 % vs 79.9 %), more participants adherent to medications having a positive experience with study medications (8.0 (2.1) vs 7.2 (1.8), range [0–10]) and significantly more participants who were non-adherent receiving UC (60.1 % vs 42.4 %) while 57.6 % of the adherent group received ADT treatment compared to 39.1 % of the non-adherent group. Table 1 shows that 2 weeks treatment response is not associated with 18 weeks of adherence.

The odds ratios (ORs) for adherent versus non-adherent to medication and the results of the logistic analysis are shown in Table 2. The treatment group to which the participant was randomized (p < 0.001) and the participant’s medication experience (p < 0.001) were significantly associated with adherence. After adjusting for medication experience, participants randomized to the ADT arm had 2.40 (95 % CI: 1.52, 3.81) times the odds of being adherent to the study medication compared to participants in the UC arm. After adjusting for the treatment group, participants with a more positive medication experience had 1.30 (95 % CI 1.14, 1.45) times the odds of being adherent. A treatment only model was also performed to ensure adjusting for the medication experience did not decrease the treatment arm effect (mediated effect) and the odds were found to be very similar (2.12 [95 % CI: 1.37, 3.28]).

The supplemental table outlines the reasons for non-adherence to medication at three different time points. The most frequently cited reasons for not taking the medication at 2, 6 and 12-week follow-ups were “Forgot, lost, or ran out of medication (64.8 %, 54.6 % and 50.6 %),” followed by “Experienced side effects (15.7 %, 27.3 % and 23.6 %),” and “Didn’t like the medication (6.5 %, 6.1 % and 5.9 %),” respectively. A small number of participants reported not using their medication because they “Quit” or “Changed their mind about quitting (2.8 %, 3.1 % and 4.2 %).”

## 4. Discussion

Over half of our participants remained compliant with their pharmacotherapy throughout the 18-week treatment period, strictly taking more than 80 % of their medication during all reporting periods. Patients who received a treatment plan adapted to their smoking cessation outcomes and those who reported a positive experience with their medication exhibited better overall compliance with their long-term pharmacotherapy.

Even though there is no generally accepted definition of what level of adherence is needed to be sufficiently effective to quit smoking, greater adherence predicts higher quit rates (Bauer et al., 2021). In one of our previous studies among Black adults, those who quit at 3 months reported very high levels of adherence to varenicline compared to participants who were still smoking (95.8 % vs 80.8 %) (Nollen, 2011). Similarly, Shiffman and colleagues (Shiffman, 2008) found using nicotine patches for at least 20 days during the first 3 weeks of treatment, tripled the quit rates compared with adherence lower than 20 days. While improving medication adherence is an important goal, it is important to recognize that its relationship with treatment outcomes is not strictly causal. Individuals who experience early treatment success may stop treatment early because they don’t feel it is needed given quitting success.

Our findings showed that participants who received an adaptive treatment were 2.4 times more likely to adhere to their prescribed medication. Switching medications is a common strategy used to increase treatment response with several chronic conditions (Almirall et al., 2014). In smoking cessation treatment, this practice remains relatively uncommon. Current tobacco treatment guidelines recommend using smoking cessation aids for 7–12 weeks, which often means continuing the same medication regardless of the patient’s response (Fiore & Baker, 2008).

Having a positive experience with the prescribed pharmacotherapy was also a strong predictor of adherence, increasing the likelihood of participants being highly compliant with their pharmacological treatment by 30%. The qualitative data also supported these findings. Similar to previous research (Minian, 2024), most individuals who were not compliant reported negative experiences with medication, such as “experiencing side effects,“ or ”disliking the medication“. However, the most common reason for non-compliance was simply forgetting to take the medication. Future studies should explore strategies to enhance adherence to smoking cessation medication, such as daily text message reminders for Black adults who smoke.

A key strength of this study is the secondary data analyses of a randomized trial that only enrolled Black adults motivated to quit smoking (Nollen, 2023), offering valuable insight into potential strategies for enhancing adherence in an often understudied population. However, the study also has some limitations. First, the current study should not be interpreted as suggesting that Black adults are the only demographic facing challenges in achieving medication adherence. Future investigations assessing factors associated with medication adherence amongst other subgroups of cigarette users would further expand the literature. Second, we only addressed characteristics associated with adherence to medication among participants who smoke daily and were interested in stopping smoking as they were eligibility criteria of the parent trial. Also, the parent trial was conducted only among Black adults in a single Midwestern city, limiting the generalizability of these findings to populations outside of the scope of this study. Moreover, using self-report data was also a potential limitation of this study. However, for the main outcome data we corroborated this information with objectively monitored medication use. Lastly, we averaged the medication beliefs across the three time points to represent their overall beliefs about the treatment interventions across the duration of the study. In conclusion, our findings identified adaptive designs and positive experiences with smoking cessation treatments as associated with adherence to smoking pharmacotherapies. Negative experiences with the prescribed medication and forgetting to take the medication were often highlighted as reasons for non-compliance. Understanding characteristics that contribute to medication adherence and reasons for nonadherence is key to informing strategies aimed at improving treatment response and, consequently, achieving smoking cessation outcomes.

## Supplementary Material

1

## Figures and Tables

**Table 1:** Baseline and follow up characteristics by Week 18 adherence.

	Non-adherent (n = 156)	Adherent (n = 177)	All (n = 333)	P value
**Baseline Demographic Characteristics**				
Age, mean (SD), y	54.0 (11.7)	53.4 (10.8	53.7 (11.2)	0.57
Sex assigned at birth, n (%)				
Female	86 (55.1)	111 (62.7)	197 (59.2)	0.16
Male	70 (44.9)	66 (37.3)	136 (40.8)	
Employed full-time or part time, n (%)	59 (37.8)	69 (39.0)	128 (38.4)	0.83
Some college or tech school, n (%)	73 (46.8)	85 (48.0)	158 (47.5)	0.82
Had health insurance, n (%)	109 (69.9)	120 (67.8)	229 (68.8)	0.68
Household income, mean (SD), $/y	25768.4 (19354.6)	22141.8 (18096.5)	23840.7 (18755.8)	0.08
Own a home	34 (21.8)	22 (12.4)	56 (16.8)	0.02
**Baseline Tobacco use Characteristics**				
Cigarettes per day (CPD) in the past 7 d, mean (SD)^1^	12.2 (7.0)	13.1 (7.2)	12.7 (7.1)	0.27
Menthol smoker, n (%)	141 (90.4)	154 (87.0)	295 (88.6)	0.33
Smoke within 30 min of waking^2^, n (%)	117 (75.0)	149 (84.2)	266 (79.9)	0.04
Urinary cotinine, mean (SD), ng/mL^3^	3409.8 (2634.4)	2913.9 (2461.3)	3146.2 (2552.1)	0.08
3-hydroxycotinine/cotinine, mean (SD), ng/mL^3^	3.7 (4.2)	3.5 (4.9)	3.6 (4.6)	0.67
Depressive symptoms, total score, mean (SD)^4,5^	5.4 (4.9)	4.8 (5.2)	5.1 (5.1)	0.23
Generalized anxiety symptoms, total score, mean (SD)^6^	5.0 (5.3)	4.1 (5.2)	4.5 (5.3)	0.11
Perceived stress^7^, mean (SD)	4.4 (3.2)	4.3 (3.6)	4.3 (3.4)	0.95
Perceived discrimination^8^, mean (SD)				0.82
Frequency of situations encountered	5.9 (5.5)	5.7 (5.3)	5.8 (5.4)	
Neighborhood disadvantage^9^, mean (SD)				0.68
Neighborhood vigilance	15.6 (5.5)	15.9 (5.5)	15.7 (5.5)	
Adverse Childhood Experiences^10^, total #, mean (SD)	2.3 (2.2)	2.4 (2.4)	2.4 (2.3)	0.47
**Follow-up Measures**				
Experience with medication,^11^ mean (SD)	7.2 (2.1)^12^	8.0 (1.8)	7.6 (2.0)	<0.001
Study arm, n (%)				
Enhanced usual care (UC)	95 (60.1)	75 (42.4)	170 (51.1)	<0.001
Adapted therapy (ADT)	61 (39.1)	102 (57.6)	163 (49.0)	
Ever use of marijuana^13^, n (%)	71 (45.5)	82 (46.3)	153 (46.0)	0.88
Ever use of Pain Medication^14^, n (%)	34 (21.8)	37 (20.9)	71 (21.3)	0.84
Ever use of Other Tobacco Products^13^, n (%)	28 (18.0)	28 (15.8)	56 (16.8)	0.60
Abstainers at 2 weeks, n (%)	49 (32.5)^15^	69 (39.0)	118 (36.0)	0.22
Any adverse event experienced^16^, n (%)	37 (23.7)	39 (22.0)	76 (22.8)	0.71

1 Assessed using a Timeline Follow-Back (TLFB) measure.

2 Assessed using a single item from the Fagerström Test for Nicotine Dependence (FTND): ’How soon after you wake up do you smoke your first cigarette? Thirty minutes or less indicates clinically significant nicotine dependence.

3 Values represent total (free/unconjugated plus glucuronide).

4 Measured with the 9-Item Patient Health Questionnaire depression screening; range, 0–27 (most severe).

5 Participants with a history of depression or anxiety with new or worsening symptoms in the last 6 months, treatment changes in the last 3 months, and those who did not feel that their symptoms were under control were excluded; participants with any suicide thoughts or attempts in past 6 months also excluded.

6 Measured with the 7-item Generalized Anxiety Disorder screening, range 0–21 (most severe).

7 Measured with 4-item Perceived Stress Scale (PSS), range 0–16 (most often).

8 Measured with the 5-item Williams experiences of discrimination short form, range 0–25 (most frequent discrimination).

9 Measured with a 6-item neighborhood vigilance questionnaire, range 6–30 (most serious problems).

10 Measured with a 10-item adverse childhood experiences questionnaire, range 0–10 (most frequent adverse experiences).

11 This was an average across time points 2,6,12. Values greater than 5 indicate a positive experience, values less than 5 indicate a negative experience.

12 3 participants were excluded as they never tried the study medication.

13 Participant use was classified if 7-day TLFB measured at baseline or any of the follow up visits (Weeks 2, 6, 12 or 18) indicated marijuana or other tobacco products use.

14 Assessed with a question adapted from the National Survey on Drug Use and Health (NSDUH) at the baseline and weeks 6, 12, and 18.

15 Five participants were missing at Week 2.

16 Any adverse event experience was measured at weeks 2, 6 and 12. Participants who reported side effects at any assessment time point were categorized as experiencing side effects.

**Table 2:** Logistic regression model predicting adherence to ≥80 % of prescribed smoking cessation medications across 18 weeks of treatment.

2-Variable Model	OR (95 % CI)	P-value

Treatment ADT vs UC	2.40 (1.2, 3.81)	<0.001
Medication experience	1.28 (1.14, 1.45)	<0.001
Treatment Only Model	OR (95 % CI)	P-value
Treatment ADT vs UC	2.11 (1.37, 3.28)	<0.001

## Data Availability

Data will be made available on request.

## Appendix A. Supplementary data

Supplementary data to this article can be found online at https://doi.org/10.1016/j.addbeh.2025.108431.
