# Supplementary material for: Characteristics associated with medication adherence in a randomized clinical trial of multiple pharmacotherapy adaptations based on treatment response in black adults who smoke
Source: Addict Behav. Author manuscript; Available in PMC 2026 May 22. (PMC13196000; doi:10.1016/j.addbeh.2025.108431)
Supplement: 1 [file NIHMS2169495-supplement-1.docx]

| Supplemental table 1: Reasons for non-adherence with the smoking cessation medication by timepoints^a^ | | | | |
| --- | --- | --- | --- | --- |
|  | Reasons, n (%) | 2-week follow-up  N = 108^b^ | 6-week follow up N =132^c^ | 12-week follow-up  N = 170^d^ |
|  | 1. Forget, lost, or ran out of study medication | 70 (64.8) | 72 (54.6) | 86 (50.6) |
|  | 1. Experienced side effect* or new medical contraindication | 17 (15.7) | 36 (27.3) | 40 (23.6) |
|  | 1. Didn’t like the medication* | 7 (6.5) | 8 (6.1) | 10 (5.9) |
|  | 1. Quit smoking and didn’t need it | ---- | 3 (2.3) | 4 (2.4) |
|  | 1. Changed my mind about quitting | 3 (2.8) | 1 (0.8) | 3 (1.8) |
|  | 1. Wanted to quit without medication | 3 (2.8) | 1 (0.8) | 5 (2.9) |
|  | 1. Personal beliefs about this medication | 1 (0.9) | 2 (1.5) | 2 (1.2) |
|  | 1. A family member, friend, health care professional or someone else expressed concern about this medication | 2 (1.9) | 2 (1.5) | 3 (1.8) |
|  | 1. Concerned about smoking on medication | 2 (1.9) | ---- | ---- |
|  | 1. Personal or family issues | 3 (2.8) | 5 (3.8) | 11 (6.5) |
|  | 1. Imposed noncompliance due to hospitalized or incarceration | ---- | 1 (0.8) | 3 (1.8) |
|  | 1. Other/No reason provided | ---- | 1 (0.8) | 3 (1.8) |
| ^a^ Counseling sessions at weeks 2, 6, and 12 included a conversation about participants experience with the medication since the last in-person visit, including barriers to taking the medication as prescribed. Following each session, counselors categorized each participants adherence status as ‘fully compliant,’ ‘partially compliant,’ ‘tried and stopped,’ and ‘never took the medication’ and the primary reason for non-adherence among those who were not fully compliant. Individuals who were partially compliant intended to take the medication but experienced barriers while those who tried and stopped actively decided to stop taking the medication.  ^b^ Among the 108 who were not fully compliant at week 2, 93 intended to take the medication as prescribed but experienced barriers (partial compliance), 15 actively made a decision to stop taking the medication (tried and stopped), and 0 never took the medication (never took)  ^c^Among the 132 who were not fully compliant at week 6, 96 intended to take the medication as prescribed but experienced barriers (partial compliance), 35 actively made a decision to stop taking the medication (tried and stopped), and 1 never took the medication (never took)  ^d^Among the 170 who were not fully compliant at week 12, 114 intended to take the medication as prescribed but experienced barriers (partial compliance), 48 actively made a decision to stop taking the medication (tried and stopped), and 8 never took the medication (never took)  *These options were not asked of patients who never took their medication (n = 1 at 6-week follow-up; n = 8 at 12-week follow-up) | | | | |
